# A systematic review of regulatory and educational interventions to reduce the burden associated with the prescriptions of sedative-hypnotics in adults treated for sleep disorders

**DOI:** 10.1371/journal.pone.0191211

**Published:** 2018-01-22

**Authors:** Elsa Bourcier, Virginie Korb-Savoldelli, Gilles Hejblum, Christine Fernandez, Patrick Hindlet

**Affiliations:** 1 Sorbonne Université, INSERM, Institut Pierre Louis d’épidémiologie et de Santé Publique, IPLESP UMR-S1136, Paris, France; 2 Service de pharmacie, Hôpital Saint-Antoine, Assistance Publique - Hôpitaux de Paris, Paris, France; 3 Faculté de pharmacie, Université Paris-Sud, Châtenay-Malabry, France; 4 Service de pharmacie, Hôpital Européen Georges Pompidou, Assistance Publique - Hôpitaux de Paris, Paris, France; Charité - Universitätsmedizin Berlin, GERMANY

## Abstract

**Background:**

The burden of Sedative-Hypnotics (SHs) has been known since the 1980s. Yet, their consumption remains high. A systematic review of the literature should help to assess efficient interventions to improve the appropriate use of SHs in sleep disorders.

**Objectives:**

To identify and assess regulatory and educational interventions designed to improve the appropriate use of SHs for insomnia treatment.

**Methods:**

We conducted a systematic review of the literature according to PRISMA guidelines. A systematic search covering the period 1980–2015 was carried out in Medline, Web of Science, Embase and PsycInfo. We included studies reporting the implementation of regulatory or educational strategies directed towards patients and/or healthcare professionals to improve the appropriate use of SHs to treat insomnia in the community, hospitals and nursing homes.

**Results:**

Thirty-one studies were included: 23 assessed educational interventions (recommendations by mail/email, computer alerts, meetings, mass media campaigns, prescription profile), 8 assessed regulatory interventions (prescription rule restriction, end of reimbursement). The most recent was implemented in 2009. Restrictive prescription rules were effective to reduce the consumption of targeted SHs but led to a switch to other non-recommended SHs. Among educational interventions, only 3 studies out of 7 reported positive results of mono-faceted interventions; whereas, 13 out of the 16 multi-faceted interventions were reported as efficient: particularly, the active involvement of healthcare professionals and patients and the spread of information through mass media were successful. The risk of bias was high for 24 studies (mainly due to the design), moderate for 3 studies and weak for 4 studies.

**Conclusion:**

Educational multifaceted studies are presented as the most efficient. But further better designed studies are needed to make evidence-based results more generalizable.

## Introduction

Sedative-hypnotics (SHs) are drugs used to treat insomnia: benzodiazepines (BZDs), z-drugs, first generation antihistamines, antidepressants being the most commonly used. These drugs were first considered as an innovation for the treatment of insomnia, but their place in the therapeutic strategy has evolved, concurrently with new findings on their efficacy and safety profile. The World Health Organization (WHO) defines the appropriate use of a drug as the ‘appropriate medicine, in doses that meet patients’ individual requirements, for an adequate period of time, and at the lowest cost both to them and the community’[1]. Yet, since the 1980s, many studies have shown that SHs were not only associated with numerous Adverse Drug Events (ADEs) but also that they had a similar or lower efficacy than non-pharmacological therapeutic strategies:

- Regarding ADEs, (defined as ‘any untoward medical occurrence that may present during treatment with a medicine but which does not necessarily have a relationship with this treatment’[2]) benzodiazepines were found to be associated, among others, with: an increased risk of falls leading to emergency visits, cognitive impairment and recently, with the onset of dementia [3–9]. According to recent studies, short half-life BZDs, z-drugs and long half-life BZDs were associated with a similar risk of falls in the elderly [10–12]. In addition, a recent report alerted to the dramatic increase in the number of zolpidem-related emergency visits for Adverse Drug Reactions (ADRs, defined as ‘a response to a drug which is noxious and unintended and occurs at doses normally used in man […]’[13]) in the USA, between 2005 and 2010 (+ 13,376 visits), challenging the fact that z-drugs were safer than other SHs [14]. Benzodiazepines and z-drugs are not the only matters of concern: first generation first generation antihistamines are also responsible for serious ADRs and ADEs such as cognitive impairment, confusion, dizziness and muscarinic effects. Their use is thus considered potentially inappropriate, especially in the elderly [15–17].
- Regarding SHs efficacy, it was found to be as or less effective than Cognitive and Behavioural Therapies (CBTs) to improve sleep parameters such as sleep onset latency, total sleep time and sleep efficiency, but inefficient at retaining therapeutic gain in the long term [18–20].

Considering these data, the current international guidelines recommend using SHs as second line treatment, when CBTs are ineffective or inappropriate. If prescribed, benzodiazepines with short half-lives, z-drugs or melatonin receptor agonists should be preferred to other drugs and prescribed at the lowest effective dosage for the shortest duration of time[21–23].

However, SH consumption remains high. Data are mainly related to community dwelling consumption. In the USA, approximately 5% of adults are treated with BZDs, with an upward trend between 2000 and 2010 [24, 25]. In France 5.6% of adults are treated with SHs [26]. In hospitals data are sparse but an initiation of SH treatment is reported for 8.2% to 33% of patients formerly untreated [27–30]. This clearly shows that the development of guidelines is insufficient and must be supported by other actions to promote the appropriate use of SHs.

To date, literature reviews addressed the global consumption of benzodiazepines without distinction as to indication. In particular, Gould *et al*. assessed interventions implemented to reduce the use of benzodiazepines in the elderly and Smith et al. assessed interventions to reduce the use of benzodiazepines in long term users (non-systematic review). These two reviews evaluated exclusively educational interventions [31, 32]. To our knowledge, there is no systematic review of the literature integrating:

- interventions to improve the use of sedative-hypnotics in the specific context of insomnia although this disorder and anxiety are two distinct conditions, with distinct treatment strategies;
- not only benzodiazepines but also all other drugs used in insomnia: first generation antihistamines and antidepressants are widely used despite their potential for ADE;
- regulatory interventions, since educational interventions are not the only way to improve the appropriate use of SH in the specific context of sleep disorders;
- interventions occurring as early as possible in the care pathway (instead of targeting long term users only) thus avoiding the development of addiction, especially with benzodiazepines.

The objective of this systematic review is thus to identify and assess regulatory and educational interventions directed toward patients and/or healthcare professionals to improve the appropriate use of SHs in sleep disorders.

## Methods

### Review protocol

This systematic review was conducted according to PRISMA (Preferred Reporting Items for Systematic reviews and Meta-Analyses) guidelines [33]. Methods used to screen the literature, select studies of interest, extract data, analyse the risk of bias and summarize findings are described below.

### Eligibility criteria

Studies meeting the following criteria were included: original articles, published in English or French language from 01/01/1980 to 10/07/2015, reporting the implementation of regulatory or educational strategies directed toward patients and/or healthcare professionals in the community, hospitals or nursing homes in view to improving the appropriate use of SHs in insomnia treatment.

Studies had to address at least one of the following outcomes: changes in prescription and/or consumption rate of SH, switch to another non-recommended medication (based on state of knowledge at the time of the study and as reported by authors), changes in healthcare resource use, clinical adverse events due to the intervention. The assessment of the intervention had to be performed: before/after the intervention and/or comparing a group that received the intervention and a control group.

Interventions reported by studies had to be compared to usual care or, in the case of multifaceted interventions to usual care or to only one intervention.

Since the objective was to have an exhaustive overview of interventions implemented between 1980 and 2015, all study designs were considered.

Interventions studied:

- Regulatory interventions: interventions led by policy makers, including ending or modification of medication reimbursement (cost sharing), formulary restrictions, specific authorization for prescribers, limited prescription duration, specific forms for prescription, pay for performance strategies and medication withdrawal from the market.
- Educational interventions: written or oral recommendations on the appropriate use of SH directed towards healthcare givers or patients or both. They could be carried out in different ways: mail or email (printed educational material), computer alerts (automatic warning sent by the computerized physician order entry for each SH prescribed), meetings, feedback after preliminary audit, prescription profile (mail with descriptive characteristics of physician’s prescriptions), visit to prescriber’s surgery (educational outreach visit) and/or mass media (television, radio…). They could be monofaceted (a single element composing the intervention) or multifaceted (interventions composed with two elements or more), tailored or not. They could be delivered by pharmacists, physicians or other healthcare givers.

Studies were excluded if interventions:

- did not focus on sleep disorders
- did not provide enough details to determine if targeted drugs were used for the treatment of insomnia
- addressed paediatric patients, psychiatric patients or prisoners.

### Information sources

On July 10^th^, 2015 we conducted a combined search in four databases: The Web of Science^®^, Medline^®^ (Pubmed), Embase^®^ and PsycINFO^®^. The reference lists of selected studies as well as articles citing selected studies were hand-screened through an iterative process, to identify potential additional studies.

### Search strategy

Our electronic search strategy combined, for each database, controlled terms (MeSH, Emtree, PsycINFO thesaurus) and natural language. Terms searched were related to interventions and sedative-hypnotic drugs. Queries for the four databases are presented in S1 Table. The strategy was approved by two librarians expert in scientific database search.

### Study selection

Eligibility of retrieved studies was independently assessed by two reviewers (EB and PH) on the basis of title, abstract and full text reading if title and abstract were not informative enough. In the case of disagreement, it was *a priori* decided that a third reviewer (CF) would be consulted to decide whether or not the study should be included. The degree of agreement between the two reviewers was assessed through the Cohen’s kappa coefficient and was considered as good if comprised between 0.60 and 0.74 and very good if equal or superior to 0.75 [34, 35]. The software RStudio^®^ version 0.98.1091 was used for this analysis.

### Data collection process

A standardized data collection grid was elaborated by EB and PH. For each selected study, EB extracted the data and completed the grid. Independently, PH checked all the data collected in the grid. In the case of disagreement, it was decided *a priori* that a third reviewer (CF) would be consulted.

### Data items

The list of data items collected for each study included in the review is presented in Table 1.

**Table 1:** Data items collected for studies included in the review.

**General information**	Journal name, title, authors’names, year of publication, country
**Study characteristics**	Design^a^ and settings
**Intervention characteristics**	Type of intervention, intervention manager, target population, target drugs, number of components of the intervention, description of each component, timing of the intervention, intervention duration, integrity of the intervention^a^
**Participant characteristics**	Number of participants^a^, sex^a^, age^a^, baseline characteristics^a^, lost to follow up^a^, drop-outs^a^, participation rate^a^
**Outcomes**	Changes in prescription and/or consumption rates of SH (regardless the unit of analysis used: percentages, defined daily doses…) Switch to another non-recommended medication (based on state of knowledge at the time of the study and as reported by authors) Changes in healthcare resource use, clinical adverse events related to the intervention
**Data collection**	Method for outcome assessment (eg. standardized or not, blinding)^a^
**Other**	Ethical committee, funding sources

^a^Data items collected in order to proceed to the evaluation of the risk of bias.

### Review update

Automated alerts were used all along the study process in order to keep the review up to date. A final update was made by one of the authors (EB) in November 2017 to cover the period 10/07/2015–20/11/2017. No supplementary article was retrieved. Therefore, results are presented according to the initial searches (period 01/01/1980–10/07/2015).

### Risk of bias in individual studies

The risk of bias in individual studies was independently assessed by two reviewers (EB and PH) using the Quality Assessment Tool for Quantitative Studies (QATQS) developed by the Effective Public Health Practice Project (EPHPP) [36]. In the case of disagreement it was decided *a priori* that a third reviewer would be solicited to reach a consensus. This tool is recommended to assess the risk of bias in studies whether they are randomized and controlled or not, which was expected to be the case in the context of this review [37]. This tool evaluates eight sources of bias: (A) selection bias, study design (B), confounders (C), blinding (D), data collection method (E), withdrawal and drop-outs (F), intervention integrity and statistical analyses (G). The risk of bias is individually rated for components A to G (strong, moderate or weak risk). Then a global rating is assigned to each study (strong, moderate or weak risk of bias).

### Summary measures

The summary of measures depends on the results reported by selected studies: when possible (more than two studies assessing the same intervention and sharing the same outcome, the same population and the same presentation of results) a meta-analysis would be performed. Otherwise, the summary of measures was presented as a descriptive synthesis.

### Risk of bias across studies

The risk of bias across studies was assessed through a graphical representation of the percentage of studies with a strong, moderate or weak risk of bias for each source of bias considered in the QATQS tool (selection bias, study design, confounders, blinding, data collection method, withdrawals and drop-outs) [36].

## Results

### Study selection

**Fig 1:**
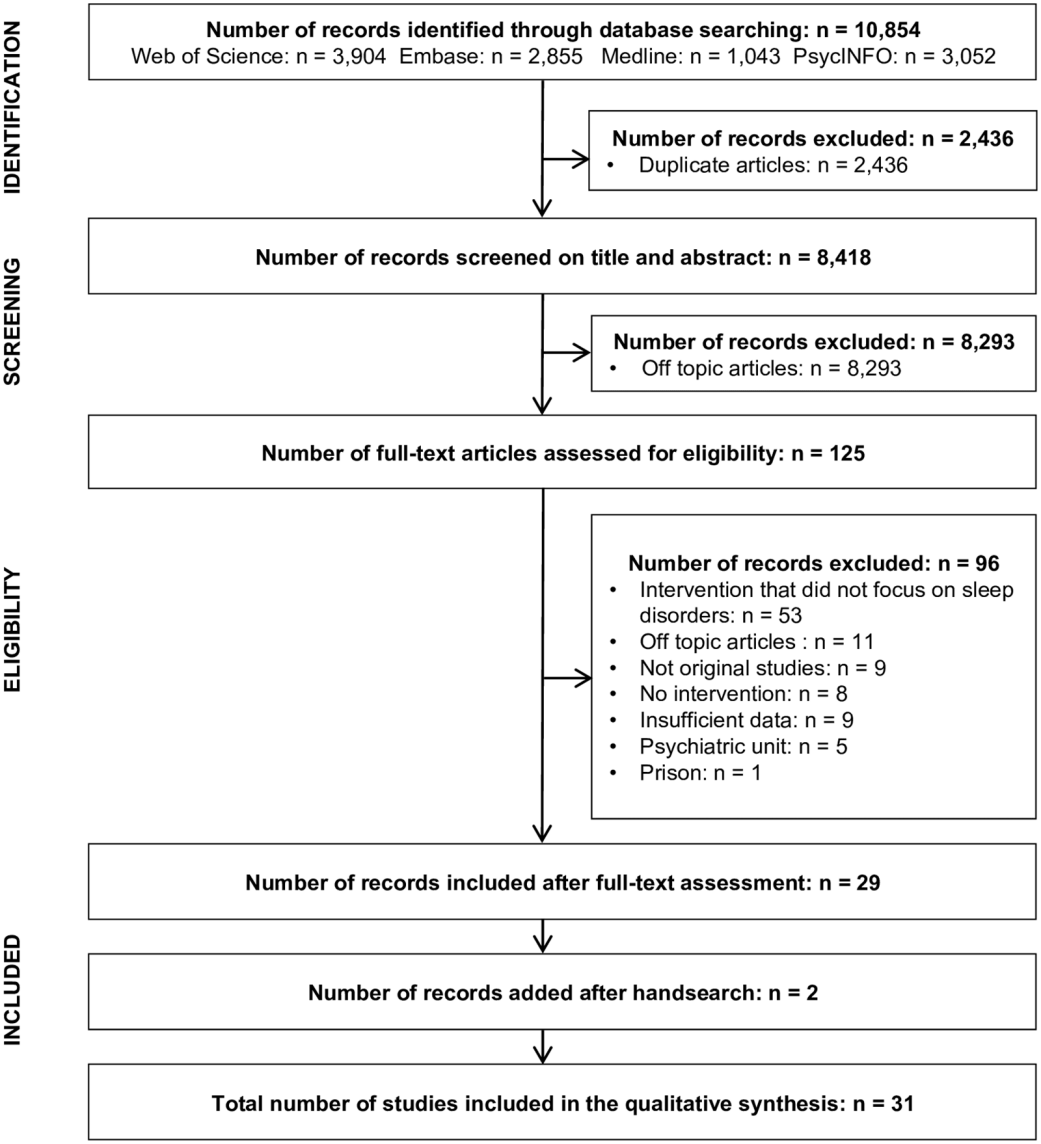
PRISMA flow diagram for the systematic review of regulatory and educational interventions to reduce the burden associated with the prescriptions of sedative-hypnotics in adults treated for sleep disorders.

Our search strategy in the four databases of interest resulted in the identification of 10,854 records. After the first round of screening, 125 records were assessed for eligibility on the basis of full-text reading. This resulted in the selection of 29 articles. The degree of agreement between the two reviewers was good (Cohen’s kappa coefficient: 0.66 [IC_95%_: 0.51–0.81]). Hand search yielded 2 additional articles. After the whole process, 31 articles were selected for the review. Details of the selection process and reasons for exclusion are presented in Fig 1.

### Study characteristics

Study characteristics are presented in S2 Table. Twenty-five studies were based on a Before-After (BA) design. The six remaining studies were multicentre Randomized Controlled Trials (RCTs) [38–43].

Twelve studies out of the 31 selected for the review were conducted in Europe (6 different countries) [29, 39, 44–53] and 11 were conducted in the United States of America [40, 42, 43, 54–61]. With 7 studies conducted in Australia and New Zealand, Oceania was also strongly involved in combating SH misuse [38, 41, 62–66]. Only 1 study was conducted in Asia [67].

All settings were represented: community, hospitals and nursing homes.

The impact of educational interventions was assessed in 23 studies: they were based on the diffusion of information about sleep hygiene and insomnia and on therapeutic recommendations through written documents, electronic supports, meetings, audio, video and poster campaigns. They targeted hypnotic benzodiazepines, z-drugs, antihistamines and antidepressants used as SHs. These interventions were developed by pharmacists and/or physicians. Seventeen studies out of 23 were directed toward health care professionals only: 10 were directed toward prescribers only [38, 39, 41, 44, 46, 54, 57, 59, 60, 66] and 7 were directed towards 2 or more different health care professionals (pharmacists, physicians, nurses) [29, 40, 42, 43, 51, 52, 63]. Two studies directly targeted patients [49, 61] and 4 targeted both health care professionals and patients [48, 62, 64, 65].

The impact of regulatory interventions such as restriction of SH prescription conditions was assessed in 8 studies [45, 47, 50, 53, 55, 56, 58, 67]. Regulatory strategies were managed by national or local authorities and were directed toward physicians. They targeted hypnotic benzodiazepines, z-drugs and antihistamines.

### Risk of bias within studies

Considering the 25 studies with a BA design, a strong, moderate, and weak risk of bias was assigned to 22, 1 and 2 studies, respectively, a distribution significantly different (P = 0.017, Fisher exact test) from that resulting from the corresponding assignments in the 6 studies based on RCTs which were 2, 2, and 2, respectively. The risk of bias assigned to each study together with the main results are shown in S3 Table.

### Results of individual studies

The heterogeneity in outcomes, interventions and results made meta-analysis impossible. Results are thus presented as a descriptive synthesis.

#### Regulatory interventions (S3 Table)

Positive results were reported in 7 out of the 8 studies assessing regulatory interventions [45, 47, 50, 55, 56, 58, 67]. Ending of reimbursement and restriction of prescription rules were presented as effective strategies to reduce the prescription and/or the consumption of targeted SHs (up to 85%). Only 2 studies assessed the onset of adverse events or switching to other non-recommended drugs that could be related to the intervention [56, 58]. Both reported a switch to other non-recommended sleep medication (histamine H1 antagonists, barbiturates).

#### Educational interventions (S3 Table)

Among the 23 studies assessing educational interventions, 7 were monofaceted and 16 were multifaceted.

Recommendations by mail or email were the most frequent strategy studied in monofaceted interventions: three studies reported negative results [59, 60, 62] and one reported negative results on prescription rates but a significant decrease of the rate of SH initiation (15.5% of hospitalised patients after the intervention *vs* 28.6% before, p < 0.001) [29]. One study assessed the impact of training seminars on the rate of prescription change by physicians: a modification was suggested for 68.4% of SH prescriptions for insomnia (n = 1045) and led to treatment cessation and dosage decrease in 5.4% and 22.8% of cases, respectively [44]. In the hospital setting, computer alerts were reported as effective for decreasing overall consumption of SHs (15% of patients after the intervention compared to 18% before, p < 0.001) but not all targeted drugs were impacted [54]. Implementation of non-pharmacologic protocols based on relaxation were also reported as effective (31% of patients treated with SHs during the intervention compared to 54% before the intervention period, p < 0.002) [61].

Multifaceted interventions assessed combinations of two or three interventions. Among the 9 interventions based on written recommendations or computer alerts combined with multidisciplinary training seminars or direct contact with healthcare givers, 5 reported significant positive results [38, 43, 51, 63, 66], one reported positive results but no statistical test supports this result [52], and 3 reported no significant change[41, 42, 64]. Among the 3 multifaceted interventions with at least one component based on recommendations through mass media, 2 reported significant positive results [49, 65] and 1 reported positive results but no statistical test supports their findings [48]. Results of studies assessing the combination of the prescription profile of the physician and written recommendations are contradictory with two negative results [39, 46] and one positive result (number of prescriptions decreased by 26.5% in the intervention group *vs* 2.9% in the control group, p = 0.004) [40].

Only 4 studies out of the 23 assessing educational interventions assessed the onset of adverse events or the switch to other non-recommended drugs related to the intervention [40, 43, 48, 65]. A worsening of sleep quality was effectively reported by one multifaceted educational intervention in a nursing home [43].

Detailed results are presented in S3 Table.

**Fig 2:**
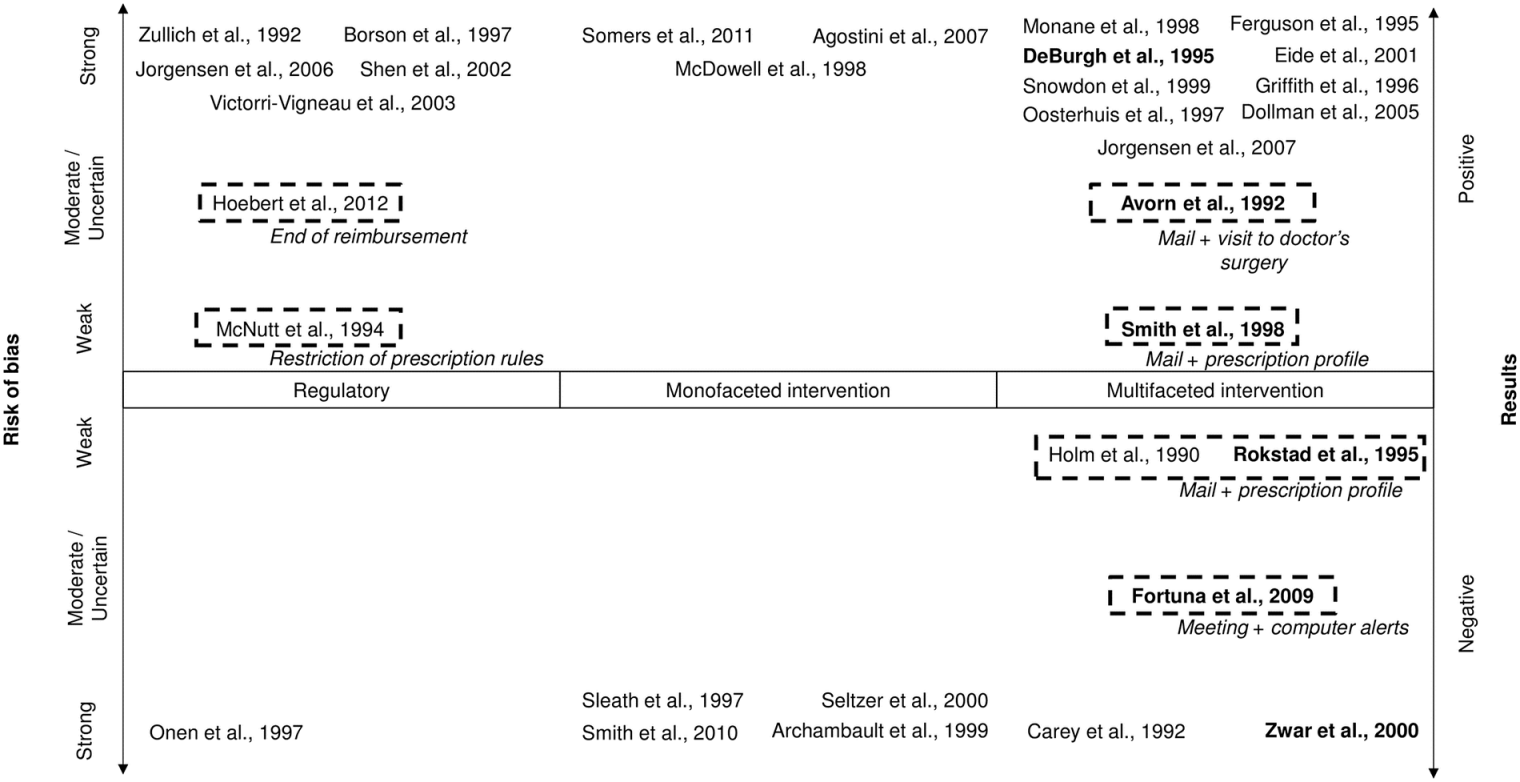
Representation of study results as a function of study design, type of intervention and risk of bias. Bold text: Randomized Controlled Trials. Italics: type of intervention.

A graphical representation of study results as a function of study design, type of intervention and risk of bias is given in Fig 2. Most studies assessing regulatory interventions reported positive results but globally, the studies on regulatory interventions had a high risk of bias. Among educational interventions, no study with low risk of bias can assert the positive results of studies assessing mass media interventions. Regarding interventions assessing the combination of written recommendations by mail with prescription profile of physicians, results are contradictory with two low risk of bias studies reporting negative results [39, 46] and one low risk of bias study reporting positive results [40].

### Risk of bias across studies

**Fig 3:**
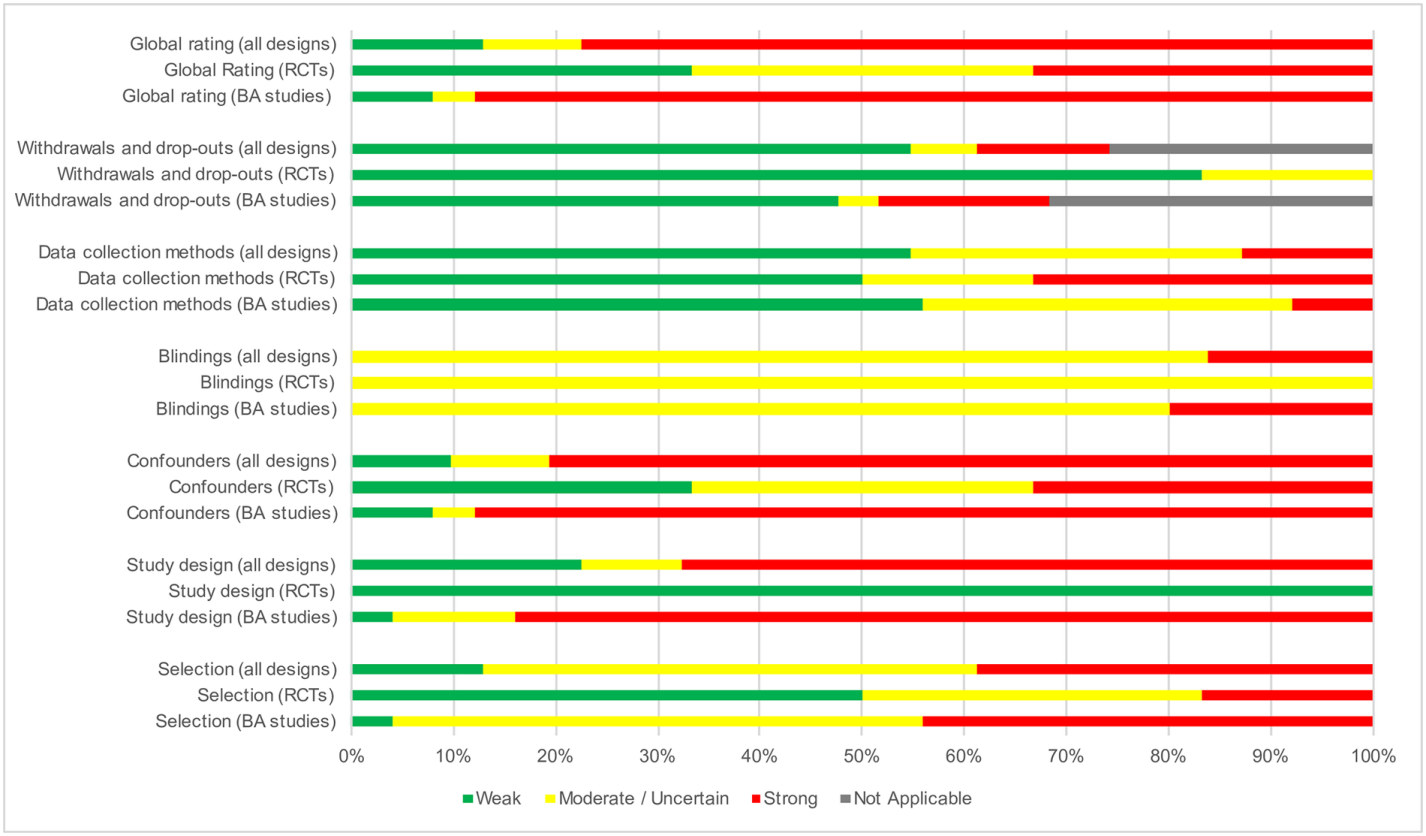
Risk of bias across studies (all designs confounded, among RCTs only and among BA studies only) according to the Quality Assessment Tool for Quantitative Studies.

Considering the QATQS, the global risk of bias is strong (high) for 77.4% of studies, moderate or uncertain for 9.7% of studies and weak for 12.9% of studies. When assessed separately, 1/3 of RCTs has a strong (high) global risk of bias, 1/3 has a moderate or uncertain risk of bias and 1/3 has a weak global risk of bias. The global risk of bias is higher in BA studies: 88% are at high risk, 4% have a moderate or uncertain risk of bias and 8% have a weak risk. Main concerns are: the presence of confounders or the absence of information regarding potential confounders, the study design and the selection bias. Details are shown in Fig 3.

## Discussion

### Summary of evidence

The objective of this systematic review was to identify and assess interventions directed toward patients and/or healthcare professionals and aimed to improve the appropriate use of SHs in sleep disorders. At the end of the screening process, 31 studies reporting interventions implemented between 1988 and 2009 were selected. Among retrieved studies, 23 assessed educational interventions and 8 assessed regulatory interventions. Regulatory interventions were based on the restriction of prescription rules and the end of reimbursement for targeted SHs. Educational interventions assessed were mono or multifaceted and were based on the diffusion of recommendations through mail or emails, computer alerts, meetings, mass media campaigns but also audit and prescription profiles.

Regarding regulatory interventions, the implementation of restrictive rules was presented as an effective way to control the consumption of SHs. However, interventions often targeted only one pharmacological class of SHs (eg. benzodiazepines), allowing prescribers and patients to switch to other classes of SHs. Only two studies assessed this phenomenon. Interestingly, both of them observed it, suggesting that the problem was displaced but not solved [56, 58]. The end of BZDs and z-drugs reimbursement implemented in the Netherlands led to a slight reduction of treatment initiation but only for new diagnosis of insomnia [45]. As suggested in a pharmaco-economic study [68], these disappointing results could be explained by the fact that in the Netherlands alternatives to BZDs are not reimbursed.

Regarding educational interventions, multifaceted interventions actively involving healthcare professionals and patients (written recommendations, phone calls, computer alerts, prescription profile associated with interactive meetings or visits to a doctor’s surgery) are the most successful strategies with 13 out of 16 studies reporting positive results. Conversely, passive monofaceted interventions (mails or emails alone) were insufficient: only 3 studies out of 7 reported positive results. The involvement of pharmacists in half of the intervention teams corroborates their central educational role at the interface of physicians/nurses and patients.

One of the particularities of these interventions is that for 22 of them (5 regulatory and 17 educational interventions) the first line target was healthcare professionals. Yet, in a context of chronic SH consumption and/or difficult access to CBTs, involving patients and getting their adherence is of prime importance for a successful outcome: two studies reported patients’reluctance [44, 60]; whereas, when interventions targeting healthcare professionals were supported by the use of media to diffuse the information to potential patients or consumers, an improvement of the appropriate use of SHs was reported [48, 65]. This phenomenon has been observed in other healthcare domains i.e., an interrupted time series study observed that repeated large-scale TV, radio and press campaigns deployed at national level and aiming at decreasing the inappropriate use of antibiotics achieved a 26.5% reduction in prescriptions (CI_95%_: -33.5% to—19.6%).

### Limitations

The results of our systematic review should be interpreted with caution and some critical points should be taken into account. When evaluating public health actions, it is of prime importance to collect outcomes allowing the assessment of health benefits, but outcomes on potential negative consequences of the intervention must also be explored. Yet, 80% of included studies did not investigate the onset of potential negative consequences of their intervention (e.g. withdrawal syndrome, switch to other non-recommended medications), and interestingly, 3 out of the 6 studies exploring unfavourable outcomes observed negative consequences [43, 56, 58]. Furthermore one-third of included studies targeted only one or two SHs, preventing the generalisation of the results on the global SH consumption in the context of insomnia treatment [40, 42, 43, 50, 52, 54–57, 62, 63, 66, 67].

In order to have an exhaustive overview, we included studies whatever the year of publication. Studies included in this review were thus conducted over a large period of time 1988–2009 and published between 1990 and 2012. This point challenges the generalisability and the transferability of results: in particular, short duration BZDs and z-drugs were considered as safer in 3 studies, and the recommendation was thus to switch long duration BZDs to short duration drugs [51, 54, 66]. Currently, considering new safety and epidemiological data, this practice is strongly discouraged, especially in the elderly [4, 11, 17, 69]. Results considered as positive in these studies could thus no longer be considered to be so. Another element that could affect the transferability of interventions assessed in this review is the lack of economic data regarding the cost of the interventions. Interventions must, indeed be cost effective so that healthcare systems can afford them. Among the 31 studies, only one indicated an extra cost of the intervention, that was planned and accepted by the local healthcare system [48].

The potential risk of bias affecting results of retrieved studies should be taken into account. The risk was rated as high for 24 studies out of 31, limiting the interpretation of three-quarters of the studies. The question of the integrity of interventions was raised for almost all studies and 2 of them actually reported a failure of the implementation of the intervention [57, 60]. In the others, data about the context and potential contamination by concomitant interventions was lacking, thus rating the risk as moderate or uncertain by the EPHPP tool, and under- or over-estimating the reality. The bias of selection present in monocentric studies, in studies based on volunteer participation or on private insurance data is also a limiting factor for the interpretation of results. Finally, since the aim of the present review was to have an overview of all interventions implemented to improve the appropriate use of SHs in insomnia, we made the choice to include studies whatever their design. Hence, 77% of the resulting studies on this subject included in this review had a non-controlled before-after design with a corresponding strong risk of bias, preventing the association of the outcome evolution observed with a specific role of the intervention under study. Experimental (RCTs) or quasi experimental designs (interrupted time series, in the case of national or regulatory interventions) with a follow-up period long enough to study the sustainability of results should be preferred.

## Conclusion

This systematic review addresses interventions aiming at improving the appropriate use of SHs in the treatment of insomnia. Despite methodological bias, multi-faceted interventions, involving healthcare professionals and patients and including health promotion initiatives through mass media are presented as the most successful strategies in retrieved studies. Difficulties encountered, especially in the interpretation of results, constitute a limiting element, but this review constitutes a useful tool for outlining some priority topics for a potential future research agenda in the domain addressed in this review. Future studies should globally be based on better designs: experimental (RCTs) or quasi experimental designs (interrupted time series, in the case of national or regulatory interventions) with a follow-up long enough to study the sustainability of results. This should enable the use of evidence-based results that are more generalizable and that can be scaled up. We also need more studies about the role of knowledge of the prescribing physician. We need more knowledge on the regulatory regulations and limitations and their particular influence on insomnia management. We need more knowledge on the role of the Internet, which has become a major information tool in many health domains but is not always well used. Importantly, several outcomes, specific to the domain investigated and yet insufficiently or not at all taken into account, should be considered in future studies: we need more information about the specific kind and phenotype of insomnia that is addressed by the interventions. We also need more knowledge on the kind and duration of SH therapy in these studies. The rates of switching to alternative non-recommended drugs, health nomadism, use of counterfeit drugs bought on the Internet, and onset of withdrawal syndrome also constitute, in our view, interesting outcomes which merit attention in future investigations.

## Supporting information

S1 Table Detailed search criteria for the Web of Science^®^, Medline^®^, Embase^®^ and PsycINFO^®^. (PDF)

S2 Table Characteristics of studies included in the review. (PDF)

S3 Table Results of individual studies and risk of bias within studies. (PDF)

S1 File PRISMA check-list. (PDF)
