# Supplementary material for: A systematic review of regulatory and educational interventions to reduce the burden associated with the prescriptions of sedative-hypnotics in adults treated for sleep disorders
Source: PLoS One. 2018 Jan 22;13(1):e0191211. doi: 10.1371/journal.pone.0191211 (PMC5777652; doi:10.1371/journal.pone.0191211)
Supplement: S1 Table — (PDF) [file pone.0191211.s001.pdf]

Supporting Table 1. Detailed search criteria for the Web of Science®.

Query number	Queries	Query number	Queries
**1**	Hypnotic*	**9**	Educat*
**2**	Sedative*	**10**	Prescri*
**3**	Antihistaminic*	**11**	« program evaluation »
**4**	Benzodiazepine*	**12**	1 OR 2 OR 3 OR 4 OR 5 OR 6 OR 7 OR 8
**5**	zopiclone	**13**	9 OR 10 OR 11
**6**	eszopiclone	**14**	12 AND 13<br>Indexes=SCI-EXPANDED, SSCI, A&HCI, CPCI-S, CPCI-SSH Timespan=All years
**7**	zolpidem	**15 = final request**	**12 AND 13**<br>**Refined by: LANGUAGES: (ENGLISH OR FRENCH) AND PUBLICATION YEARS: (2014 OR 2003 OR 1995 OR 1981 OR 2012 OR 2005 OR 1992 OR 1987 OR 2013 OR 2002 OR 1991 OR 1985 OR 2011 OR 1998 OR 1993 OR 1984 OR 2010 OR 1999 OR 1990 OR 1980 OR 2009 OR 1994 OR 1989 OR 2008 OR 2000 OR 1986 OR 2007 OR 2001 OR 1988 OR 2006 OR 1996 OR 1982 OR 2004 OR 1997 OR 1983 OR 2015)**<br>**Timespan: All years. Indexes: SCI-EXPANDED, SSCI, A&HCI, CPCI-S, CPCI-SSH.**
**8**	zaleplon		

SCI-EXPANDED: *Science Citation Index Expanded*; SSCI: *Social Science Citation Index*; A&HCI: *Art and Humanities Citation Index*; CPCI-S: *Conferencee Proceedings Citation Index-Science*; CPCI-SSH: *Conference Proceedings Citation Index-Social Sciences & Humanities*; « * »: truncation.

Supporting Table 1 (continued). Detailed search criteria for Medline® (Pubmed).

Query number	Queries
**1**	"Hypnotics and Sedatives"[Mesh]
**2**	"Histamine H1 Antagonists"[Mesh]
**3**	"Benzodiazepines"[Mesh]
**4**	"zaleplon" [Supplementary Concept]
**5**	"zolpidem" [Supplementary Concept]
**6**	"zopiclone" [Supplementary Concept]
**7**	"eszopiclone" [Supplementary Concept]
**8**	hypnotic*[tw]
**9**	sedative*[tw]
**10**	antihistaminic*[tw]
**11**	benzodiazepine*[tw]
**12**	zaleplon [tw]
**13**	zolpidem [tw]
**14**	zopiclone [tw]
**15**	eszopiclone [tw]
**16**	"Education, Professional"[Mesh]
**17**	"Physician's Practice Patterns"[Mesh]
**18**	"Program Evaluation"[Mesh]
**19**	"Drug Utilization Review"[Mesh]
**20**	1 OR 2 OR 3 OR 4 OR 5 OR 6 OR 7 OR 8 OR 9 OR 10 OR 11 OR 12 OR 13 OR 14 OR 15
**21**	16 OR 17 OR 18 OR 19
**22**	20 AND 21
**23 = final query**	**20 AND 21 Filters: Publication date from 1980/01/01 to 2015/12/31; English; French**

Mesh: *Medical Subject Headings* (controlled terms, in green); tw: *Text Word* (natural language, in violet); *Supplementary concept*: controlled terms for chemicals; « * »: truncation.

Supporting Table 1 (continued). Detailed search criteria for Embase®.

Query number	Queries	Query number	Queries
**1**	'hypnotic sedative agent'/exp/mj AND [embase]/lim	**6**	educat* AND [embase]/lim
**2**	'histamine h1 receptor antagonist'/exp/mj AND [embase]/lim	**7**	1 OR 2
**3**	'clinical practice'/exp AND [embase]/lim	**8**	3 OR 4 OR 5 OR 6
**4**	'prescription'/exp AND [embase]/lim	**9 = final query**	**7 AND 8 AND ([english]/lim OR [french]/lim) AND [1980-2015]/py**
**5**	'program evaluation'/exp AND [embase]/lim		

exp: explosion; mj: *major topic*; lim: limit, « * »: truncation; in green: EMTREE language; in violet: natural language.

Supporting Table 1 (continued). Detailed search criteria for PsycINFO®.

Query number	Queries
**1**	MJSUB.EXACT.EXPLODE(« Hypnotic Drugs »)
**2**	MJSUB.EXACT.EXPLODE(« Antihistaminic Drugs»)
**3**	MJSUB.EXACT.EXPLODE(« Benzodiazepines»)
**4**	hypnotic*
**5**	sedative*
**6**	antihistaminic*
**7**	benzodiazepine*
**8**	zopiclone
**9**	eszopiclone
**10**	zolpidem
**11**	zaleplon
**12**	MJSUB.EXACT.EXPLODE(« Program Evaluation»)
**13**	MJSUB.EXACT.EXPLODE(« Clinical Practice»)
**14**	educat*
**15**	prescri*
**16**	1 OR 2 OR 3 OR 4 OR 5 OR 6 OR 7 OR 8 OR 9 OR 10 OR11
**17**	12 OR 13 OR 14 OR 15
**18**	16 AND 17
**19 = final query**	**16 AND 17 AND la.exact("English" OR "French") AND pd(>19800101)**

MJSUB: *Major Subject Headings*; pd: *Publication Date*; la: *language*; « * »: truncation. In green: controlled language; in violet: natural language.
