# Supplementary material for: A systematic review of regulatory and educational interventions to reduce the burden associated with the prescriptions of sedative-hypnotics in adults treated for sleep disorders
Source: PLoS One. 2018 Jan 22;13(1):e0191211. doi: 10.1371/journal.pone.0191211 (PMC5777652; doi:10.1371/journal.pone.0191211)
Supplement: S2 Table — (PDF) [file pone.0191211.s002.pdf]

Supporting Table 2. Characteristics of studies included in the review.

Settings	Type of intervention	Study	Country	Study design	Targeted drug(s)	Leader of the intervention	Professionals or population targeted	Component(s) of the intervention	Beneficiary population
Community	Regulatory	McNutt et al., 1994 [55]	USA	BA, cohort, EPIC program	Triazolam, Temazepam, Flurazepam	State	Physicians	Specific prescription form for BZDs in 1989	Elderly
		Hoebert et al., 2012 [44]	Netherlands	BA, multicentre	Hypnotic and anxiolytic BZDs, z-drugs	State	Physicians + patients	BZDs ending of reimbursement in 2009	Adults (all ages)
		Jorgensen et al., 2006 [46]	Denmark	BA, multicentre	Hypnotic BZDs, z-drugs	State	Physicians	Hypnotic prescription by telephone forbidden, prescription duration ≤ 30 days	Adults (all ages)
		Victorri-Vigneau et al., 2003 [49]	France	BA, (Social security cohort)	Flunitrazepam	State	Physicians	Specific prescription form in 2001,	Adults (all ages)
	Educational	Monane et al., 1998 [56]	USA	BA, cohort (MMMC program)	Flunitrazepam	Pharmacists	Physicians	Computer alert triggering phone call to physicians by a pharmacist to discuss therapeutic alternative	Elderly
		Archambault et al., 1999 [43]	France	BA, multicentre	Hypnotic BZDs, z-drugs	Physicians + Pharmaceutical firm	Physicians	2 training sessions, application on 5 patient cases	Adults (all ages)
		De Burgh et al., 1995 [37]	Australia	RCT, multicentre	BZDs used as hypnotics	Physicians or pharmacists	Physicians	Visit to doctor's surgery, written documents for physicians and patients, audio and video tapes	Adults (all ages)
		Dollman et al., 2005 [64]	Australia	BA, multicentre	BZDs used as hypnotics	Research team + sleep expert	Physicians + pharmacists + nursing home professionals + patients	Written recommendations for all + training sessions for physicians and pharmacists + multidisciplinary meetings for prescriptions + radio campaign + poster	Adults (all ages)
		Ferguson et al., 1995 [65]	New Zealand	BA, multicentre	Temazepam, triazolam	Physicians and pharmacists	Physicians	Prescription profile addressed to physicians + meeting on insomnia to promote triazolam replacement with temazepam	Adults (all ages)
		Holm, 1990 [45]	Denmark	BA, multicentre	Hypnotic BZDs, z-drugs	Research team (physicians)	Physicians	Meeting or written information + prescription profile or no intervention	Adults (all ages)
		Oosterhuis et al., 1997 [48]	Netherlands	BA, cohort	All drugs taken to treat insomnia	Educational channel (TV)	General population	8 TV lessons on insomnia with guidance for CBT + 9 additional radio lessons + book and audio material on demand	Adults (all ages)

Supporting Table 2 (continued). Characteristics of studies included in the review.

Settings	Type of intervention	Study	Country	Study design	Targeted drug(s)	Leader of the intervention	Professionals or population targeted	Component(s) of the intervention	Beneficiary population
Community	Educational	Rokstad et al., 1995 [38]	Norway	RCT, multicentre	All drugs taken to treat insomnia	Research team (physicians)	Physicians	Written guidelines and prescription profile	Adults (all ages)
		Seltzer et al., 2000 [58]	USA	BA (Medicaid cohort)	Hypnotic BZDs, barbiturates	Texas Medicaid drug committee (physicians + pharmacists)	Physicians	Written guidelines sent to physicians presenting inappropriate prescriptions	Adults (all ages)
		Sleath et al., 1997 [59]	USA	BA (Medicaid cohort)	All drugs taken to treat insomnia	New Mexico Medicaid program	Physicians	Written guidelines sent to physicians presenting inappropriate prescriptions	Adults (all ages)
		Smith et al., 1998 [39]	USA	RCT, multicentre	Triazolam, temazepam, estazolam, quazepam, flurazepam	Washington Medicaid Program + pharmaceutical research team	Physicians and/or pharmacists	Prescription profile et recommendations for physicians presenting inappropriate prescriptions	Adults (all ages)
		Zwar et al., 2000 [40]	Australia	RCT, multicentre	All BZDs used to treat insomnia	General practitioners (research team)	Trainee general practitioners	Visit to general practitioner's surgery + written guidelines for GP and patients (intervention group) or intervention on another subject (control group)	Adults (all ages)
		Jorgensen et al., 2007 [47]	Denmark	BA, multicentre	Hypnotic BZDs, z-drugs	Medical advisor	Physicians + patients	Elimination of hypnotic prescription by telephone, prescription duration ≤ 30 days + meeting with physicians, pharmacists and nursing home professionals + written information and poster for physicians and patients + article in the newspaper	Adults (all ages)

Supporting Table 2 (continued). Characteristics of studies included in the review.

Settings	Type of intervention	Study	Country	Study design	Targeted drug(s)	Leader of the intervention	Professionals or population targeted	Component(s) of the intervention	Beneficiary population
Hospital	Regulatory	Shen et al., 2002 [66]	Taïwan	BA (Medicaid cohort)	Flunitrazepam	State	Physicians	Specific form + authorisation + written recommendations for flunitrazepam prescription (oct. 2000), restriction of prescription duration for non-psychiatrists	Adults (all ages)
		Onen et al., 1997 [52]	France	BA, monocentre	BZDs, zolpidem, zopiclone and associations of hypnotics	State	Physicians	Decree restricting hypnotic prescriptions duration	Elderly
	Educational	Agostini et al., 2007 [53]	USA	BA, monocentre	Diphenhydramine, diazepam, lorazepam, trazodone	Physicians via a computer order-entry system	Physicians	Warning screens with recommendation for alternative pharmacological (trazodone) or non-pharmacological therapy for each prescription of diazepam or diphenhydramine	Elderly
		Griffith et al., 1996 [51]	UK	BA, monocentre	Temazepam	Physicians + Pharmacists + nurses	Physicians and nurses	Development of guidelines + information meetings + written recommendations for residents	Elderly
		McDowell et al., 1998 [60]	USA	BA, monocentre	BZDs, histamine H1 receptor antagonists	Nurses	Patients	Alternative non-pharmacological procedure when patients asked for hypnotics (hot drink, relaxation, massages) + written information given at patient's discharge	Elderly
		Carey et al., 1992 [63]	Australia	BA, monocentre	All drugs prescribed to treat insomnia	Pharmacists + physician specialized in addictions	Physicians + pharmacists + nurses + patients	Physicians: information meetings,<br>Nurses: 2 information meetings at the beginning + weekly meeting,<br>Patients: video on the hospital TV channel + written information + relaxation	Adults (all ages)
		Fortuna et al., 2009 [41]	USA	RCT, multicentre	Ambien® (zolpidem), Lunesta® (eszopiclone), Sonata® (zaleplon), Rozerem (ramelteon)	Physicians (research team)	Physicians + nurses	Computer alert (prescription of zolpidem or trazodone generic instead of targeted drugs + link to educational material) or computer alert + information meeting or no intervention	Adults (all ages)
		Somers et al., 2011 [28]	Belgium	BA, monocentre	BZDs, z-drugs, trazodone, tricyclic antidepressants, H1 receptor antagonists	Drug committee (physicians + pharmacists)	Physicians + nurses	Written guidelines + e-mail with the same guidelines.	Adults (all ages)

Supporting Table 2 (continued). Characteristics of studies included in the review.

Settings	Type of intervention	Study	Country	Study design	Targeted drug(s)	Leader of the intervention	Professionals or population targeted	Component(s) of the intervention	Beneficiary population
Nursing homes	Regulatory	Borson et al., 1997 [54]	USA	BA, multicentre	Triazolam, temazepam, flurazepam, diphenhydramine	State	Physicians	Law and guidelines for sedative-hypnotics appropriate use (1987, 1989, 1992)	Elderly
		Zullich et al., 1992 [57]	USA	BA, multicentre	Hypnotic BZDs	State	Physicians	Specific form for BZD prescriptions (1989)	Elderly
	Educational	Avorn et al., 1992 [42]	USA	RCT, multicentre	Diphenhydramine	Geriatrician + pharmacists	Physicians + Nurses	Physicians: 3 information letters + 3 meetings; Nurses: 4 meetings; Control group: no intervention	Elderly
		Eide et al., 2001 [50]	Norway	BA, multicentre	Diazepam, oxazepam, nitrazepam, flunitrazepam, zopiclone, zolpidem mianserin, alimemazine, promethazine, levomepromazine,	Pharmacists	Physicians + nurses + nursing home directors	Written + oral report to present the results of a previous audit and recommendations on SHs appropriate use	Elderly
		Smith et al., 2010 [61]	Australia	BA, multicentre	Temazepam, nitrazepam	Pharmacists (research team)	Physicians + pharmacists + nurses + patients	3 emails with recommendations on SHs appropriate use	Elderly
		Snowdon et al., 1999 [62]	Australia	BA, multicentre	Temazepam, nitrazepam	State	Physicians + pharmacists	Working group on psychotropic drugs + meetings for physicians + grant for pharmaceutical consultations	Elderly

BA: Before-After study; BZDs: Benzodiazepines; E: Educational; EPIC: Elderly Pharmaceutical Insurance Coverage; MMMC: Merck-Medco Managed Care; RCT: Randomized Controlled Trial; R: regulatory; SHs: Sedative-Hypnotics.
