# Supplementary material for: A systematic review of regulatory and educational interventions to reduce the burden associated with the prescriptions of sedative-hypnotics in adults treated for sleep disorders
Source: PLoS One. 2018 Jan 22;13(1):e0191211. doi: 10.1371/journal.pone.0191211 (PMC5777652; doi:10.1371/journal.pone.0191211)
Supplement: S3 Table — (PDF) [file pone.0191211.s003.pdf]

Supporting Table 3. Results of individual studies and risk of bias within studies.

Type of intervention		Study	Results	Adverse events / switch to another non-recommended medication	Global risk of bias
Regulatory	Ending of reimbursement	Hoebert *et al*., 2012 [44]	**Number of hypnotic BZDs and z-drugs initiation for 100 insomnia diagnosis in 2009 compared to 2008:** 59.1% *vs* 67.0% ($p < 0.05$). No influence of the gender. No more statistically significant difference for patients aged 75 and older after stratification on the age.	Not investigated	Moderate
	Restriction of prescription rules	Onen *et al*., 1997 [52]	**Evolution of the index [number of SH administration per year/number of days of hospitalization per year] in 1992-1994 compared to 1990-1991*:** BZDs : 0.06 *vs* 0.13; Zolpidem & Zopiclone: 0.23 *vs* 0.00; associations: stable; global consumption: 0.34 *vs* 0.20.	Not investigated	High
		Shen *et al*., 2002 [66]	**Number of flunitrazepam prescriptions between October 2000 and January 2001 compared to the period between June and September 2000:** Δ = - 46.0% (ns). **Number of flunitrazepam prescriptions between February and May 2001 compared to the period between June and September 2000:** Δ = - 63.9% ($p < 0.05$) (complete cessation for only 5% of patients).	Not investigated	High
		Borson *et al*., 1997 [54]	**Number of patients receiving SHs nightly in 1992 compared to 1989:** Triazolam: Δ =-85.8% ($p < 0.001$); Temazepam: Δ = + 4.5% (ns); Flurazepam: na; Diphenhydramine: Δ =-45.4% ($p = 0.001$). **Number of patients receiving SHs only « as required » in 1992 compared to 1989:** Triazolam: Δ = 85.7% ($p < 0.001$); Temazepam: Δ = + 70.8% ($p = 0.01$); Flurazepam: Δ =-66.6% ($p = 0.015$); Diphenhydramine: Δ =-18.6% (ns).	Not investigated	High
		Zullich *et al*., 1992 [57]	**Number of patients treated with SHs 6 months after the intervention compared to the 6 months before*:** Δ = -83.1% (complete treatment cessation for 27% of patients).	switch to chloral hydrate, diphenhydramine or phenobarbital for 73 % of patients).	High
		McNutt *et al*., 1994 [55]	**Number of HS prescriptions during the 4th quarter of 1989 compared to the 2nd quarter of 1988*:** Triazolam: Δ =-43.2%; Temazepam: Δ =-42.4%; Flurazepam: Δ =-50.4%.	Increase in the prescription of over the counter SHs: hydroxyzine: Δ = + 69 %.	low
		Jorgensen *et al*., 2006 [46]	**Evolution of the defined daily dose for 1000 patients during the 1st quarter of 2005 compared to 2003 (same intervention in the 2 centres)*:** Centre 1: BZDs = -91.5%; z-drugs = -77.8%. Centre 2: BZDs = -24.1% (2 patients treated); z-drugs: -71.8%.	Not investigated	High
		Victorri-Vigneau *et al*., 2003 [49]	**Evolution of the number of patients treated with flunitrazepam*:** January-February 2001: Δ = -41%; February-March: Δ = + 18.9%; March-September: Δ = -30%.	Not investigated	High

Supporting Table 3. (continued):

Type of intervention		Study	Results	Adverse events / switch to an other non-recommended medication	Global risk of bias
Educational	Mail or email	Smith *et al.*, 2010 [61]	**Percentage of residents treated with SHs in April 2008 compared to September 2007:**<br>**Rural setting:** 1 SH: 22% *vs* 25% (ns); ≥ 2 SHs: 2% *vs* 3% (ns).<br>**Metropolitan setting:** 1 SH: 23% *vs* 25% (ns); ≥ 2 SHs: no change : 2% (ns).	Not investigated	High
		Somers *et al.*, 2011 [28]	**Percentage of hospitalized patients treated with SHs in 2009 compared to 2000:** 43.3% *vs* 45.2% (ns).<br>**Percentage of hospitalized patients with an initiation of SHs in 2009 compared to 2000:** 15.5% *vs* 28,6% (p < 0.001).	Not investigated	High
		Seltzer *et al.*, 2000 [58]	**Percentage of prescriptions modified after the intervention*:** 40.4 % (on 234 inappropriate prescriptions).<br>**Percentage of patients with SH cessation one year after the intervention (when prescribers had planned to change the prescription)*:** 47.4%	Not investigated	High
		Sleath *et al.*, 1997 [59]	**Actions planned by physicians (126 answers on 269 inappropriate prescriptions)*:** dose decrease or cessation: 16%; advice: 55%; no action (patient reluctant): 26%.<br>**Results at 3 months (dose decrease or cessation)*:** 49% of patients for whom a change was planned; 40% of patients for whom no change was planned initially; 35% of patients for whom the physician didn't answer.	Not investigated	High
	Training seminars	Archambault *et al.*, 1999 [43]	**Prescription modification planned by physicians after the intervention (on 1,045 prescriptions)*:** yes = 68.4%; no = 31.6%.<br>**Results (on 1,045 prescriptions)*:** SH cessation = 5.4 %; Dose decrease = 22.8.	Not investigated	High
	Computer alerts	Agostini *et al.*, 2007 [53]	**Percentage of patients treated with SHs 12 months post-intervention compared to the 12 months before:** All SHs: 15% vs 18% (OR = 0.82 [$CI_{95\%}$: 0.76-0.87], p < 0.001); significant decrease (p < 0.001) for diphenhydramine and lorazepam; no change for diazepam; significant increase (p < 0.001) for trazodone.	Not investigated	High
	Non-pharmacologic protocol	McDowell *et al.*, 1998 [60]	**Percentage of patients treated with SHs after intervention compared to a similar population before the intervention:** 31% vs 54% (p < 0.002).	Not investigated	High
	Computer alert + phone call	Monane *et al.*, 1998 [56]	**Percentage of prescriptions changed after intervention:** 40% (on 1679 alerts) compared to a theoretical percentage of change estimated at 2% without intervention (p<0.001).	Not investigated	High

Supporting Table 3. (continued):

Type of intervention		Study	Results	Adverse events / switch to an other non-recommended medication	Global risk of bias
Educational	Written recommendations or computer alerts or mail + meetings or visit to doctor's surgery	Carey *et al.*, 1992 [63]	**Percentage of SH initiations 3 months post intervention compared to 3 months before:** 15.4% *vs* 13.8% (ns).	Not investigated	High
		Fortuna *et al.*, 2009 [41]	**Adjusted risk ratio of prescription:** Usual care: RR = 1.31 [CI95%: 1.08-1.60]; Computer alerts: RR = 0.97 [CI95%: 0.82-1.14]; Computer alert + meeting: RR = 0.98 [CI95%: 0.83-1.17].	Not investigated	Moderate
		Ferguson *et al.*, 1995 [65]	**Evolution of the number of triazolam and temazepam prescriptions (recommendation = switch triazolam to temazepam) between the period August-October 1988 and the period August-October 1989:** Triazolam = - 40% and temazepam = + 21.6% ($p < 0.001$).	Not investigated	High
		DeBurgh *et al.*, 1995 [37]	**Percentage of SH prescriptions per 100 insomnia diagnosis 5 months post compared to 2 months pre-intervention:** IG: 87.4% *vs* 94.5%; CG: 88.5% *vs* 92.4% (ns)<br>**Percentage of SH initiation for new insomnia diagnosis per 100 encounters 5 months post compared to 2 months pre-intervention:** IG: 48.3% *vs* 84.7%; CG: 64.3% *vs* 68.9%; OR associated to the intervention = 0.21 [CI95%: 0.05 0.80]	Not investigated	High
		Zwar *et al.*, 2000 [40]	**Percentage of SH prescriptions per 100 insomnia diagnosis:**<br>IG: before intervention 70.5%; 6 months after 70.3%; 12 months after 66.0%.<br>CG: before intervention 77.2%; 6 months after 70.1%; 12 months after: 73.5%<br>Difference between IG and CG: ns	Not investigated	High
		Eide *et al.*, 2001 [50]	**Percentage of patients treated with SH in 2000 compared to 1995:** All SHs: 24.1% *vs* 23.4% (ns); Nitrazepam: 8.9% vs 29.8% ($p < 0.05$); zopiclone: 53.3% vs 8.5% ($p< 0.01$); other SHs: no change.	Not investigated	High
		Avorn *et al.*, 1992 [42]	**Percentage of patients treated with diphenhydramine 30 days after intervention compared to 30 days before:** IG: 5.8% *vs* 9.1%; CG: 12% *vs* 12.2% ($p < 0.05$).	Deterioration of sleep quality (OR = 1.8; CI95%: [0.8 – 3.9])	Moderate
		Snowdon *et al.*, 1999 [62]	**Percentage of patients treated with nitrazepam and temazepam in 1998 compared to 1993:** nightly treatment: 17.0% *vs* 26.6% ($p < 0.001$); "as required" treatment: 4.8% *vs* 12.6% ($p < 0.001$).	Not investigated	High
		Griffith *et al.*, 1996 [51]	**Evolution of the number of SH tablets given per month, 6 months after the intervention compared to 6 months before*:** Δ = - 69.3%.<br>**Evolution of the number of SH prescriptions at discharge per month, 6 months after the intervention compared to 6 months before*:** Δ = - 48.0%	Not investigated	High
	Written recommendations + prescription profile	Holm *et al.*, 1990 [45]	**Evolution of the number of ddd prescribed between January and May 1989:** (A) Meeting alone: Δ = - 15.0% (ns); (B) written recommendations + prescription profile: Δ = - 6.8% ($p < 0.05$); (C) No intervention: Δ = - 16.7%.	Not investigated	Low

Supporting Table 3. (continued):

Type of intervention		Study	Results	Adverse events / switch to an other non-recommended medication	Global risk of bias
Educational	Written recommendations + prescription profile	Rokstad *et al.*, 1995 [38]	**Percentage of SH prescriptions per 100 encounters for insomnia in November 1989 compared to November 1988*:** IG: 98.4% *vs* 98.2%; CG: 98.9% *vs* 97.9%.<br>**Prescriptions of medium and long acting BZDs between November 1988 et November 1989:** IG: significant decrease; CG: no change.<br>**Prescriptions of short acting BZDs, antidepressant and histamine H1 antagonists (as advised) between November 1988 and November 1989:** IG: significant increase; CG: no change.	Not investigated	Low
		Smith *et al.*, 1998 [39]	**Evolution of the number of SH prescriptions 3 months post intervention compared to the 3 months before:** IG: Δ = -26.5%; CG: Δ = -2.9% ($p = 0.004$).	No	Low
	Mass media program	Oosterhuis *et al.*, 1997 [48]	**Evolution of the number of SH consumers (325 participants), period intervention vs period pre-intervention:** Δ = - 40% ($p < 0.01$).<br>**Evolution of the number of SH consumers, 4.5 months post-intervention compared to 4.5 months before:** Δ = - 36% ($p < 0.01$).	Not investigated	High
	Written recommendation + mass media program	Dollman *et al.*, 2005 [64]	**Evolution of the number of SH dispensation (ddd per 1000 per day) during the period November-April 2001 compared to the period November-April 1999:** Flunitrazepam: Δ = - 39% ($p < 0.05$); Nitrazepam: Δ = - 33% ($p < 0.05$); Temazepam: Δ = - 11% (ns); Alprazolam: Δ = - 28% ($p < 0.05$); Diazepam: Δ = -11% (ns); Oxazepam: Δ = - 19% ($p < 0.05$).	No	High
		Jorgensen *et al.*, 2007 [47]	**Evolution of the average number of SH prescriptions*:** BZDs: - 48.6% (min = - 26.0%; max = - 72.2%); z-drugs: - 46.4% (min = - 16.4%; max = - 64.5%).	No	High

BZDs: Benzodiazepines; CG: Control Group; IG: Intervention Group; min: minimum; max: maximum; ns: non-significant; OR: Odds Ratio; SHs: Sedative-Hypnotics; *no statistical analysis performed for these data.
